# Improving the Medical and Surgical Out-of-Hours Handover at a Hospital in Regional New South Wales, Australia

**DOI:** 10.7759/cureus.27613

**Published:** 2022-08-02

**Authors:** Stacey J Law, Steffan T Seal, Chosita Cheepvasarach

**Affiliations:** 1 Medicine, Gosford Hospital, Gosford, AUS; 2 Internal Medicine, Central Coast Local Health District, Gosford, AUS; 3 General Medicine, Royal North Shore Hospital, Sydney, AUS

**Keywords:** after-hours care, safe patient handover, continuity of patient care, patient safety, quality improvement

## Abstract

Introduction: Effective handover between shifts is widely accepted as essential for continuity of care and patient safety. Problems with out-of-hours handover were identified at our hospital, having come to light following attendance at handover meetings by the authors.

Methods: Consultation of junior doctors was performed to identify issues with the out-of-hours handover and a baseline audit was conducted to objectively assess handover practice. Local guidelines were used to create a handover tool, which was subsequently implemented and assessed via multiple PDSA (plan, do, study, and act) cycles. In addition, registrar education was undertaken. Concurrently, meetings with senior clinicians and managers were held to address wider issues including venue, intensive care registrar attendance, emergency call procedures, and implementation of an electronic handover tool.

Results: Junior doctor consultation and baseline audit identified failings in handover. Following our intervention, improvements were demonstrated in the handover of patient information, including diagnosis (50% increase), investigations (76% increase), and plan (33% increase). Doctor attendance and punctuality also improved, along with a more punctual start time and reduced handover duration of five minutes on average.

Conclusion: Bringing structure and leadership to an informal and inconsistent handover system using simple and well-defined methods can improve the quality and consistency of handover. The sustainability of the intervention was demonstrated with continued improvements seen in a subsequent cycle.

## Introduction

Care of hospital inpatients operates on a shift-based system; therefore, an inherent challenge is the safe transfer of care from daytime to out-of-hours teams. This is particularly relevant when multiple shift changes occur in a 24-hour period as there are multiple handovers, and it has been shown that retention of information across these handovers is poor [1].

Failings in a handover system can have a direct impact on patient safety [2-4]. Failure to handover effectively is a major preventable cause of patient harm and is principally due to poor communication and systemic error [5]. Failure of handover is neither a new nor isolated issue. Reports of unstructured, informal, and error-prone processes have previously been documented in an Australian public hospital [6].

Changing work patterns in recent years has emphasised the importance of good handover. Recognition of the adverse effects of doctor fatigue on patient care led to shorter working hours, and as a result, increased shift changes and frequency of handovers [7]. It is recognised that there is a lack of education around handover, with variable practice in operation and a lack of research and policy [8]. Handover is known to be highly variable and dependent upon human factors [9]. Therefore, standardisation of handover is vital for improving efficiency and patient safety. National guidelines suggest proformas to standardise verbal handover and avoid information omissions and unhelpful tangents [5-7].

Good handover practice should assign leadership, standardise the order of proceedings, use a system of communication such as ISBAR (a structure for communicating information using the following format: identification, situation, background, assessment, and recommendation), prioritise tasks, and document these elements well [7]. In addition, handover time should be protected from excessive disruption, with punctuality and professionalism promoted [7].

Our hospital is a public hospital based in a regional area of New South Wales, Australia. The hospital is a secondary care centre with approximately 500 inpatient beds. Out-of-hours cover is provided by a team of eight doctors in the evening and five overnight, covering 20 wards in total. Each shift is staffed with two registrars and a combination of interns, residents, and senior residents. The out-of-hours handover involves daily meetings at shift changes, namely, at the morning and evening shift changes. The purpose of the out-of-hours handover is to highlight unwell or deteriorating patients and allow continuity of care via the handover of outstanding jobs.

As junior doctors attending handover, we were concerned about the unstructured and informal format, often lacking leadership, which had the potential to adversely affect patient care. Attendance and punctuality were erratic, and the quality of information handed over was variable. We noted interruption of patient handover due to small groups of doctors talking amongst themselves, inconsistent handover of patient background information, and frequently a lack of patient identifiable information. Often, fewer than three patient identifiers were communicated as explicitly stated in local guidelines, and, in some instances, patients were referred to only by their location, e.g., "the lady in bed X in ward X". There was no system in place to guide or structure handover, resulting in poor handover quality and efficacy. The handover was conducted in a secured room, therefore, protecting patient confidentiality.

The primary aim of this quality improvement (QI) project was to improve the quality, efficacy, and consistency of handover at our hospital. We aimed to create sustainable change with the ultimate goal of improving out-of-hours patient care and safety.

## Materials and methods

Data collection

Qualitative data were collected via feedback from doctors, and quantitative data were collected during observation of the handover meeting. The data were collected by the authors during their participation in the handover as members of the usual medical team. Statistical analysis was completed using Microsoft Excel (Microsoft Corporation, Redmond, WA) to determine the mean, median, and percentiles.

Inclusion and exclusion criteria

Inclusion criteria consisted of out-of-hours medical and surgical handover, which occurred simultaneously, during shift change between day and night teams. Exclusion criteria were nil.

Study design

Baseline qualitative data were obtained via feedback forms distributed to doctors (19 respondents) attending handover over a two-week period. Local guidelines were consulted to determine handover requirements and identify data points for audit. Following this, eight consecutive handover meetings were observed (50 patients), and data were collected on attendance, start time, duration, and leadership. Handover structure was also recorded, including whether rapid responses were discussed first (see Table 1 for definitions). Patient-specific information handed over was recorded, including the use of three patient identifiers, the reason for handover, and background information (as per ISBAR format), including diagnosis, present condition, past medical history, investigations, plan, and resuscitation status.

**Table 1:** Definitions

Term	Definition
ISBAR	An acronym for the process of conveying patient information: introduction, situation, background, assessment, and recommendation
Intern	Medical staff who are post-graduate year one and currently in their internship
Resident medical officer (RMO)/senior resident medical officer (SRMO)	Medical staff who are postgraduate year 2+ but are yet to start a training program
Junior medical officer	Medical staff of all grades apart from consultants. Synonymous with junior doctor grades in the United Kingdom
Rapid response	A patient demonstrating imminent clinical deterioration as indicated by scoring in the "red zone" of the New South Wales (NSW) Health between the flags (BTF) observation scoring system. This requires immediate attendance of designated medical and nursing staff including senior medical and intensive care doctors
Clinical review	A patient demonstrating potential clinical deterioration as indicated by scoring in the "yellow zone" of the between the flags (BTF) observation system, requiring review by designated medical staff within one hour
Clinical aggression response team (CART) call	A patient demonstrating a threat to themselves or others due to behavioural and/or mental health deterioration requiring immediate attendance of designated medical staff

**Figure 1:**
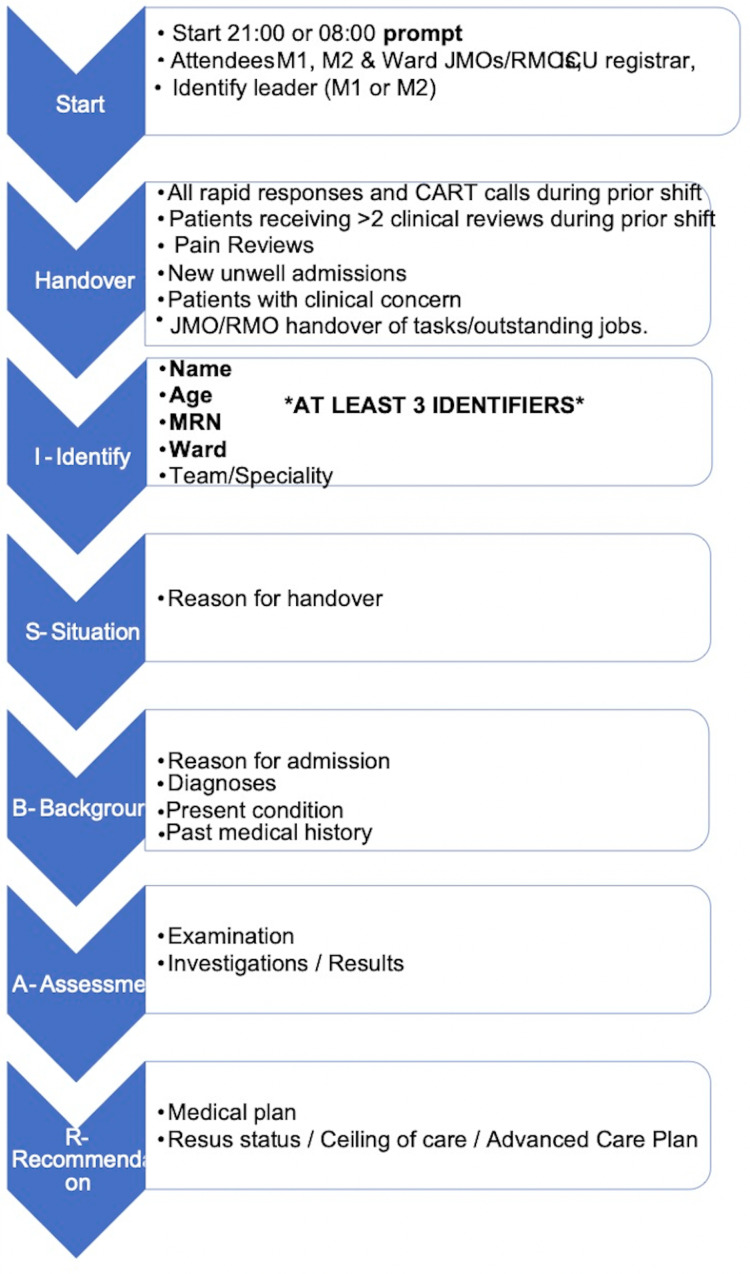
Handover tool JMO: junior medical officer; RMO: resident medical officer; CART: clinical aggression response team; MRN: medical record number.

Local guidelines (Table 2) describe the handover procedure in detail and were used as a framework to develop the handover tool (Figure 1), which was then implemented into practice in the handover meetings in two PDSA (plan, do, study, and act) cycles. The Standards for Quality Improvement Reporting Excellence (SQUIRE) guidelines [10] were adhered to throughout the design of this project.

**Table 2:** Local out-of-hours handover guidelines CCLHD: Central Coast Local Health District; CNS2: clinical nurse; JMO: junior medical officer.

Step 1	Attendees: Gosford leader – doctor M1 or M2 attendees – ICU registrar, medical doctors, M1, M2, night medical doctors, and CNS2 night duty
Step 2	Time: 2100 or 0800 sharp
Step 3	Location: Gosford: Level 2 CCLHD Conference Centre Seminar Room 2
Step 4	Equipment access to PowerChart via computer
Step 5	Patients to be handed over: Weekday all rapid responses that occurred between 1630 and 2100. All patients receiving >2 clinical reviews between 1630 and 2100. Patients reviewed by previous shift staff and subsequently uploaded to eMH. Weekend all rapid responses that occurred between 0800 and 2100. All patients receiving >2 clinical reviews between 0800 and 2100. Patients reviewed by previous shift staff and subsequently uploaded to eMH. Patients uploaded to eMH by day teams on Friday. Patients uploaded to eMH by day teams on weekend ward rounds
Step 6	Minimum patient data set: eMH is used to confirm the patient’s name, date of birth, medical record number, ward, and consultant. Minimum three patient identifiers
Step 7	Patient results: PowerChart will be open to review patient information
Step 8	Handover: Handover is to be presented clearly and systematically, for example, as per the ISBAR system and includes (where appropriate) provisional/known diagnosis, relevant medical/social history, investigations, and management plan (including resuscitation status)
Step 9	Clarification: JMOs are provided with a plan to ensure continuity of care. JMOs are supported by the consultant leader to clarify any information

The handover tool we implemented consisted of a simple proforma outlining the structure handover was expected to follow. This included rapid responses and clinical aggression response team (CART) calls to be discussed first (see Table 1), followed by clinical reviews, pain reviews, new unwell admissions, patients of clinical concern, and finally handover of jobs to junior medical staff. The tool also included the designation of a leader, patient information to be handed over, and the ISBAR format. The handover tool (Figure 1) was designed to be a user-friendly interpretation of local guidelines.

Strategy: PDSA cycle 1 (implementation of the handover tool)

As the handover is to be led by registrar grade doctors, it was important to highlight our proposed changes to this group in particular. Registrar engagement was sought directly at each handover meeting. We also communicated with all junior and senior registrars via email to highlight the issues identified and promote the use of the developed tool. A poster was created and displayed along with the handover tool in the handover room. The tool was also uploaded onto the junior doctor intranet page "JMO Central", where it could be easily accessed. Following the implementation of the tool, the authors continued to attend handover and repeated the measurements taken at baseline. A further eight consecutive sessions were observed four weeks later (42 patients) and the same data points were recorded with the tool in use.

Following each handover, junior doctors and registrars were consulted informally to gather opinions regarding the tool. Feedback was positive overall and it was mentioned that improved structure and content of the meeting were noticed with the tool in use. Constructive feedback indicated that using less text in the tool would make it more user-friendly.

Local guidelines stipulated that an intensive care unit (ICU) registrar and clinical nurse (CNS2) should attend; however, baseline measurement showed that this did not occur. We highlighted this issue to senior clinicians and ICU, and nursing teams were consulted subsequently. The handover venue was subsequently identified as an issue as it was situated far from ICU, thus drawing senior staff away from ICU and potentially affecting the safety of ICU patients. In light of this, a venue change closer to the ICU was proposed.

On two occasions during our audit, a rapid response (see Table 1 for definitions) was triggered. As per guidelines, all attendees left the handover to attend, resulting in the abandonment of the handover meeting. The deteriorating patient group was consulted and agreed that disruption of handover to this extent had implications for patient safety. It was agreed that the guideline be amended to require only one registrar and one junior doctor to attend rapid responses occurring during handover, thus allowing handover to continue with the remaining attendees.

Another concern raised during meetings with senior managers was the number of clinical reviews (see Table 1 for definitions) being requested by nursing staff for pain management. We, therefore, incorporated patients requiring clinical review for pain into the handover tool for the next cycle.

Strategy: PDSA cycle 2 (streamlining the handover tool and addition of pain reviews)

The handover tool was shortened as per feedback from attendees in PDSA 1, and the handover of pain reviews was included. Further consecutive handover sessions were observed (eight handovers, 46 patients), and measurements were taken, as in the previous cycle.

Registrar engagement continued to be sought via direct communication in the meeting; however, we noted that with a rotation change following the completion of the audit cycle, the new team were not aware of the issues or the tool in use. We, therefore, decided to repeat email communications and also address new trainees in their induction at the start of each rotation to inform them of the issues with the handover, the use of the handover tool, and how to access it.

Issues raised during further meetings with senior managers were a lack of electronic documentation and accountability for tasks handed over. Senior managers, therefore, commenced discussions with the IT department to develop and implement an electronic handover tool.

## Results

Results of the pre-intervention survey revealed that most (n = 11, 58%) did not believe handover was well structured. The three most common issues raised were inconsistent leadership (n = 10), poor attendance/punctuality (n = 9), and multiple simultaneous conversations occurring during handover (n = 3). Suggested changes included a more standardised structure (n = 4), a designated leader (n = 4), and improved punctuality/attendance by attendees (n = 3).

**Figure 2:**
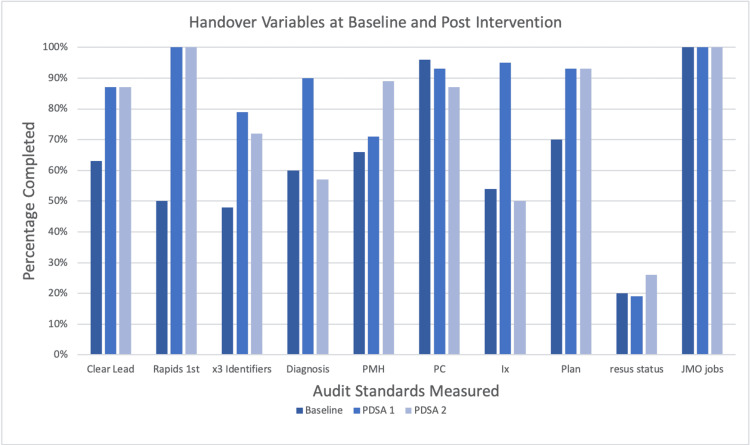
Baseline vs. post-intervention results PDSA: plan, do, study, and act cycle; Rapid: rapid response medical emergency calls; PMH: past medical history; PC: presenting complaint; Ix: investigations; Resus status: resuscitation status; JMO: junior medical officers.

Baseline measurements revealed that handover started seven minutes late on average. A clear lead was identified 63% of the time, three patient identifiers were used less than half of the time (48%), and resuscitation status was communicated only 20% of the time. The handover of background information in the ISBAR system ranged from 54% to 70%. Six doctors were absent and 10 were late across the eight observed handover meetings. See Table 3 and Figure 2 for full results.

**Table 3:** Combined results showing baseline, post-PDSA cycle 1, and PDSA cycle 2 results PDSA: plan, do, study, and act cycle; JMO: junior medical officer.

Variable	Baseline	PDSA 1	PDSA 2
Patients	50	42	46
Start time late (average)	9 min	4 min	2 min
Length (average)	17 min	12 min	12 min
Absent	6	3	5
Late	10	7	3
Clear lead	63%	87%	87%
Rapid responses first	50%	100%	100%
x3 identifiers	48%	79%	63%
Diagnosis	60%	90%	57%
Medical history	66%	71%	89%
Present condition	96%	93%	87%
Investigations	54%	95%	50%
Plan	70%	93%	93%
Resuscitation status	20%	19%	26%
JMO jobs	100%	100%	100%
Pain reviews	N/A	N/A	25%

Improvements including attendee punctuality (30% fewer doctors late), attendance (50% fewer doctors absent), identification of a leader (38% improvement), and use of three patient identifiers (65% improvement) were demonstrated in PDSA 1 when compared with baseline. Handing over of rapid responses first improved from 50% to 100%. There was a reduction in the length of handover from a mean of 17 minutes to 12 minutes and handover started an average of five minutes earlier.

Improvements were seen in the handover of patient information including diagnosis (50% increase), investigations (76% increase), and plan (33% increase). There was minimal or no change seen in the handover of past medical history, present condition, and resuscitation status.

PDSA 2 showed further improvements in attendee punctuality with 70% fewer doctors late and start time seven minutes earlier than baseline. Improvements were seen in the handover of past medical history (35% increase) and resuscitation status (30% increase) compared with baseline.

Sustained improvements were seen in the reduction of handover length, which remained five minutes shorter than baseline. Sustained improvement was also seen in leadership, handover of rapid responses first, and handover of the plan, which remained the same as in PDSA 1. Handover of three patient identifiers fell slightly from 79% to 72% but was still considerably improved compared with baseline (48%).

Improvements were not maintained in the handover of diagnosis, present condition, and investigations. Handover of pain reviews was added in cycle 2 but this was only handed over 25% of the time. See Table 3 and Figure 2 for results.

## Discussion

Results indicate that implementation of our handover tool and education of doctors improved the handover process at our hospital in terms of structure, leadership, professionalism, and information handed over.

Sustained improvements were observed from baseline in the identification of a leader, following a defined structure, handover of three patient identifiers, and handover of background information. In addition, attendance and punctuality of doctors also improved.

Unexpected improvements include handover starting an average of seven minutes earlier and being five minutes shorter in duration. Whilst this cannot be directly explained by the handover tool, it is possible that the use of the tool introduced structure and improved efficiency by reducing distractions and unnecessary interruptions. This finding is supported by studies at other centres, which showed a reduced handover length correlated with reduced distractions after the implementation of a handover agenda [11].

Handover of pain reviews was added in PDSA 2; however, this was only handed over 25% of the time. As this clinical information is not usually handed over, the poor uptake of its introduction could be due to unfamiliarity. In future cycles, further education could be employed before re-evaluating this variable.

Commonly identified issues by doctors in our pre-intervention survey included leadership, attendance, structure, and professionalism. Our results indicate that the use of the handover tool helped improve these areas with a likely subsequent improvement in patient safety. Informal feedback confirmed that doctors found the handover tool a useful adjunct for guiding structure, content, and efficiency. A post-intervention survey would have helped formalise and quantify these opinions and is something that could be implemented in future cycles.

Other quality improvement projects in the literature have demonstrated improvements in handover following the implementation of a standard operating protocol [11-14]. Studies have not yet determined a direct link to improved patient outcomes, and this is perhaps an area for further study.

Although our project focused on the role of registrars as leaders in the out-of-hours setting for handover, it could be argued the problems identified here were due to an absence of more senior medical staff. Indeed, the important role of consultants in handovers has been noted before [12]. Perhaps the important role of senior medical staff in clarifying and displaying appropriate behaviours for handover could be emphasised further.

Limitations

Although improvement of handover implies improved patient safety, this was not ratified in our study with objective data on patient outcomes. Investigation of patient safety may have been accomplished via objective markers such as the number of clinical reviews, rapid responses, morbidity, and mortality rates. These variables were not included as outcome markers in this study but would be a valuable consideration for future projects.

It is possible that reduced distractions played a role in the improved start time and shorter duration of the handover but this was not measured formally. In future cycles, we wish to identify and quantify the number of interruptions and distractions occurring during handover.

This study could also be improved by noting how unwell patients were and correlating this with variables investigated, such as length of handover, for example, as handing over more unwell patients could reasonably be assumed to take longer.

Guideline changes regarding rapid responses occurring during handover were discussed and agreed to in principle during meetings with senior management; however, this had not come into effect at the time of writing. Similarly, meetings were held with ICU and nursing groups, and a change of venue was proposed; however, this had not come to fruition at the time of writing. This demonstrates how procedural and broad changes can be slow to implement. We aim to assess the effect of these changes in future cycles.

Although implementation represents a significant challenge, further improvement in handover is likely to be achieved by the implementation of an electronic handover tool [15,16]. As this was not implemented at the time of writing, the effects of this intervention could not be analysed. Despite this, the use of modern information technology systems such as this in the context of a fully digital hospital medical documentation system would certainly be of interest.

The effectiveness of changes seen in this project will continue to be monitored by senior managers and clinicians, primarily through the district Junior Medical Officers (JMO) Quality and Safety Committee. Indeed, this committee's primary focus is the inclusion of JMOs in quality improvement. Through dual involvement of senior and cycling junior staff, the project will maintain relevance to current practice.

## Conclusions

This project demonstrates that bringing structure and leadership to an informal and inconsistent handover process using simple techniques can improve the quality and content of the handover. The sustainability of the intervention by continued application in practice was demonstrated with improvements seen in subsequent cycles. The methods used in this project are easily reproducible and may be tailored to local guidelines in other centres. This project highlights the importance of aligning clinical practice with local guidelines. This study demonstrates that junior doctor engagement with senior managers early on in the quality improvement process is essential, as this is likely to elicit greater impact and sustainability, particularly when overarching guideline change is required and multiple departments are involved.

Improvement of the handover process at our hospital remains a work in progress. It is envisaged that further improvements will be achieved via the implementation of broader strategies including guideline alteration, venue change, ICU attendance, and the creation of an electronic handover tool. The next steps include the implementation of these strategies and assessment via further quality improvement cycles and potential application to other teams/specialities within the hospital.
